# Fine Mapping and Functional Analysis of the Multiple Sclerosis Risk Gene CD6

**DOI:** 10.1371/journal.pone.0062376

**Published:** 2013-04-24

**Authors:** Bhairavi Swaminathan, Angélica Cuapio, Iraide Alloza, Fuencisla Matesanz, Antonio Alcina, Maria García-Barcina, Maria Fedetz, Óscar Fernández, Miguel Lucas, Teresa Órpez, Mª Jesus Pinto-Medel, David Otaegui, Javier Olascoaga, Elena Urcelay, Miguel A. Ortiz, Rafael Arroyo, Jorge R. Oksenberg, Alfredo Antigüedad, Eva Tolosa, Koen Vandenbroeck

**Affiliations:** 1 Neurogenomiks Laboratory, University of the Basque Country (UPV/EHU), Leioa, Spain; 2 Department of Immunology, University Medical Center Hamburg-Eppendorf, Hamburg, Germany; 3 Instituto de Parasitología y Biomedicina “López Neyra” Consejo Superior de Investigaciones Científicas (CSIC), Granada, Spain; 4 Servicio de Genética, Hospital de Basurto, Bilbao, Spain; 5 Department of Neurology, Institute of Clinical Neurosciences, Hospital Regional Universitario Carlos Haya, Málaga, Spain; 6 Unidad de Esclerosis Múltiple, Hospital Virgen Macarena, Sevilla, Spain; 7 Research Laboratory, Institute of Clinical Neurosciences, Hospital Regional Universitario Carlos Haya, Málaga, Spain; 8 Área de Neurociencias, Instituto de Investigación Sanitaria Biodonostia, San Sebastián, Spain; 9 Servicio de Neurología, Unidad de Esclerosis Múltiple, Hospital Donostia, San Sebastián, Spain; 10 Immunology Department H. Clínico S. Carlos, Instituto de Investigación Sanitaria S. Carlos (IdISSC), Madrid, Spain; 11 Multiple Sclerosis Unit, Neurology Department H. Clínico S. Carlos, Instituto de Investigación Sanitaria S. Carlos (IdISSC), Madrid, Spain; 12 Department of Neurology, University of California San Francisco, San Francisco, California, United States of America; 13 Servicio de Neurología, Hospital de Basurto, Bilbao, Spain; 14 IKERBASQUE, Basque Foundation for Science, Bilbao, Spain; Institute Biomedical Research August Pi Sunyer (IDIBAPS) - Hospital Clinic of Barcelona, Spain

## Abstract

*CD6* has recently been identified and validated as risk gene for multiple sclerosis (MS), based on the association of a single nucleotide polymorphism (SNP), rs17824933, located in intron 1. CD6 is a cell surface scavenger receptor involved in T-cell activation and proliferation, as well as in thymocyte differentiation. In this study, we performed a haptag SNP screen of the *CD6* gene locus using a total of thirteen tagging SNPs, of which three were non-synonymous SNPs, and replicated the recently reported GWAS SNP rs650258 in a Spanish-Basque collection of 814 controls and 823 cases. Validation of the six most strongly associated SNPs was performed in an independent collection of 2265 MS patients and 2600 healthy controls. We identified association of haplotypes composed of two non-synonymous SNPs [rs11230563 (R225W) and rs2074225 (A257V)] in the 2^nd^ SRCR domain with susceptibility to MS (*P*_max(T) permutation_ = 1×10^−4^). The effect of these haplotypes on CD6 surface expression and cytokine secretion was also tested. The analysis showed significantly different CD6 expression patterns in the distinct cell subsets, i.e. – CD4^+^ naïve cells, *P* = 0.0001; CD8^+^ naïve cells, *P*<0.0001; CD4^+^ and CD8^+^ central memory cells, *P* = 0.01 and 0.05, respectively; and natural killer T (NKT) cells, *P* = 0.02; with the protective haplotype (RA) showing higher expression of CD6. However, no significant changes were observed in natural killer (NK) cells, effector memory and terminally differentiated effector memory T cells. Our findings reveal that this new MS-associated CD6 risk haplotype significantly modifies expression of CD6 on CD4^+^ and CD8^+^ T cells.

## Introduction

Multiple sclerosis (MS) is a disorder of the central nervous system that is characterized by chronic inflammation, demyelination, axonal loss and neurodegeneration [1]. Genome-wide association (GWAS) screens and meta-analyses have enabled identification of about 50 non-HLA MS risk genes [2]–[16]. Apart from the HLA region, the implicated genes exert modest effects at the population level with odds ratios (OR) ranging from 1.1–1.3 [17]. In a recent study, we validated the association of four risk single nucleotide polymorphisms (SNPs) with MS susceptibility [5], in a northern Spanish-Basque population, from which the *CD6* SNP rs17824933 emerged with a stronger risk (OR = 1.34) [18].

CD6 is a member of the group B scavenger receptor cysteine-rich super family (SRCR-SF) [19] found on thymocytes, mature T-cells, some B-cell and natural killer (NK) subsets and is also expressed in certain parts of the brain like the cerebellum, basal ganglia, thalamus, corpus amygdaloideum, and cerebral lobi [20]–[23].

At the transcriptional level, in addition to the full-length form, a total of six isoforms have been reported that diversify the cytoplasmic domains [24], [25]. The ligand for CD6 is the Activated Leukocyte Cell Adhesion Molecule (ALCAM), found in the thymic epithelium and in the epithelial layer of the blood-brain barrier. ALCAM binds to the third SRCR domain of CD6 and this interaction was recently shown to enable transmigration of CD4^+^ T lymphocytes across the blood-brain barrier [26].

Functional studies using mAbs to CD6 have demonstrated its role in T cell activation, proliferation [27]–[30] and in regulating the expression of intracellular phosphoproteins and production of proinflammatory cytokines [31]. Furthermore, CD6 is involved in the maturation of the immunological synapse (IS) by associating at the central supramolecular activation cluster (cSMAC) region [25]. However, an isoform lacking the ALCAM binding domain, CD6ΔD3 which was upregulated upon T cell activation, was not localized at the IS [25], [32]. Studies using thymocytes show an increased expression of CD6 on thymocytes in the mature (single-positive) compared to the immature (double-negative, double-positive) stages and showed the negative influence of CD6 on the rate of apoptosis implicating its role in thymocyte selection [20]. The CD6ΔD3 expression was also observed to be higher on mature compared to immature thymocytes [25].

A recently published [33] correlation analysis of rs17824933 genotypes with expression of two CD6 extracellular domain isoforms (full-length and CD6ΔD3) on CD4^+^ and CD8^+^ T cells from healthy donors revealed no significant differences. However, comparison of the relative expression of the two isoforms showed the risk allele (rs17824933^G^) to be associated with a higher expression of the isoform lacking the ligand binding domain (CD6ΔD3).

In the present study, we performed a two-stage CD6 SNP screen to identify the putative causative variant(s) or haplotypes that contribute to increased MS susceptibility. We analyzed the effect of associated haplotypes on the cell surface expression of CD6 in T and NK cells by flow cytometry and assessed differences in proliferation and cytokine production (IFN-γ and IL-17) according to haplotype.

## Materials and Methods

### Sample Collections

The details of the sample collections used for the genetic study are listed in Table 1. All affected individuals meet established diagnostic criteria [34], [35], and the blood samples were obtained after written informed consent from all donors, and with the approval of the institutional ethics research committees from Bilbao (Comité Ético de Investigación Clínica (CEIC) del Hospital de Basurto), San Sebastián (CEIC del Hospital de Donostia), Madrid (CEIC del Hospital Clínico San Carlos-IdISSC), Málaga (CEIC del Hospital Regional Universitario Carlos Haya), Sevilla (CEIC del Hospital Universitario Virgen Macarena), Granada (CEIC del Hospital Universitario Virgen de las Nieves), and UCSF, USA (UCSF Institutional Review Board). Initial screening was performed using the northern Spanish-Basque dataset, which includes samples collected from two centers - the Hospital de Basurto (Bilbao) and the Donostia Hospital (San Sebastián). The replication sample sets included 2265 MS patients and 2600 healthy controls from three different collections – Andalucía (Hospital Virgen Macarena, Sevilla; Hospital Carlos Haya, Málaga; Hospital Clínico, Hospital Virgen de las Nieves and Blood Bank, Granada), Madrid (Hospital Clínico S Carlos) and University of California, San Francisco (UCSF).

**Table 1:** Clinical characteristics of the MS patients included in the genetic study.

	NORTHERN SPANISH-BASQUE				REPLICATION DATASET					
	BILBAO		SAN SEBASTIÁN		UCSF WHITES		ANDALUCÍA		MADRID	
	Controls	Cases	Controls	Cases	Controls	Cases	Controls	Cases	Controls	Cases
**Participants**										
Total number	565	573	249	250	450	507	1340	1119	810	639
% Male/Females	31.2/68.8	26.5/73.5	34/66	38/62	32.9/67.1	32/68	27.3/62.7	26.8/62.5	45.4/53.2	34.7/62.8
/Unknown	/0	/0	/0	/0	/0	/0	/0	/10.7	/1.4	/2.5
**Disease course (%)**										
RR & SP/PP/PR	–	87.6/10/0	–	88/2/0/	–	79.7/3.1/0.4/16.8/0	–	82.2/1/0.3 0.5/15.9	–	83.3/9.7/0.1
/other/no details		/1.6/0.8		/0.8/9.2						/0.5/6.4
**Age at Onset**										
Mean ± S.D.	–	30.5±10.10	–	31.6±10.3	–	33.3±9.3	–	29.4±9.9	–	28.9±8.9
**EDSS**										
Mean ± S.D	–	3.08±2	–	3.91±2.6	–	2.04±1.96	–	3.2±1.8	–	2.9±2.3

*Abbreviations*: RR = Relapsing-remitting, SP = Secondary progressive, PP = Primary progressive, PR = Primary relapsing, S.D. = Standard deviation.

### SNP Selection and Genotyping

*CD6* haplotype-tagging (haptag) SNPs were selected using the multimarker tagger algorithm from the HapMap website on the CEU population (*r^2^* cut-off 0.8; MAF 0.2; HapMap Release #27). A total of a thirteen haptag SNP were selected for the study that included nine intronic SNPs, three force-included non-synonymous SNPs and the rs17824933 reported earlier. A recently reported SNP, rs650258, located near the *CD6* gene which was found to be strongly associated with MS susceptibility [17], was also included. Genotyping of the thirteen haptag SNPs was done via CEGEN (http://www.cegen.org/primera.php?que=presentacio&lang=cast) using Sequenom technology on the northern Spanish-Basque dataset (example of genotype cluster plot in Figure S1). The Taqman genotyping kits (Life Technologies, Carlsbad, CA) for the SNPs rs11230548 (ABI custom assay, Forward/Reverse Primer Sequence: CTAACTTGCTTGGCTAAGGTGTTG/CCACAAGATACATGTTAATTACAAAGGAGGAA, Reporter 1/2 Sequence: TCTGCTAGATTTATCTGCTG/CTGCTAGATTTCTCTGCTG), rs17824933 C_33967506_10, rs916811 C_2553017_1, rs11230559 C_26898776_10, rs11230563 C_31727142_10) were used with the ABI 7900HT (Life Technologies, USA) in the datasets of Andalucía, Madrid and the UCSF whites, except for the rs2074225, which was done using the genotyping services of CEGEN or through sequencing. rs650258 was genotyped using the Taqman assay kit C_2260876_10 in all the collections. Power was calculated using the CATS power calculator (http://www.sph.umich.edu/csg/abecasis/CaTS) [36]. The genotyping success rate for all SNPs was above 95%, except for the rs916811 and rs11230563 in the Madrid collection that had success rates of 94% and 94.5% respectively.

### Statistical Analysis of Genetic Data

The data obtained was analyzed using PLINK software (version 1.07) http://pngu.mgh.harvard.edu/purcell/plink/ [37]. Hardy-Weinberg equilibrium (HWE) test was performed to check for deviances among the control population. The strength of association was assessed via the odds ratio (OR) values in the individual datasets. The Cochran-Mantel-Haenszel (CMH) test was done on the replication and combined datasets to test for association after stratification. A test of heterogeneity (Breslow-Daystest) was performed on the replication and combined datasets to identify any heterogeneity. Haplotype analysis for the original sample set was performed using the Haploview software (version 4.2) [38], and the sliding window analysis and Max-T permutation *P* values were calculated using PLINK.

### Samples for Fluorescence-activated Cell Sorting (FACS) and ELISA

10 ml fresh lithium heparinised blood was obtained by venipuncture from twenty-seven MS patients (Department of Neurology, Institute of Clinical Neurosciences, Hospital Regional Universitario Carlos Haya, Málaga, Spain) and from twelve healthy donors (University Klinikum Eppendorf, Hamburg, Germany). The clinical characteristics of these twenty-seven MS patients are listed in Table S1. Peripheral blood mononuclear cells (PBMC) were purified using a Ficoll-Hypaque gradient, as described in the supplier's protocol (ICN Biomedicals Inc., OH, USA), counted and immediately used or cryopreserved in the presence of dimethyl sulphoxide 10% (v/v) (DMSO), and 20% fetal bovine serum and stored at −196°C until further use. When using frozen samples, PMBC were thawed immediately before use.

### Flow Cytometry

The list of monoclonal antibodies (mAb) and their suppliers are provided in the Table S2. Table S3 gives the details of the combination of markers used to identify the different cell subsets. CD3 and CD56 were used to identify T (CD3^+^ CD56^−^), NK (CD3^−^ CD56^+^), and NKT (CD3^+^ CD56^+^) cell subsets. Within T cells, CD4 and CD8 mark T-helper and T-cytotoxic subsets. Subsequently, CD45RA, CD27 and CD28 were used to distinguish naïve (CD45RA^+^ CD27^+^ CD28^+^), central memory cells (CD45RA^−^ CD27^+^) and effector memory cells (CD45RA^−^ CD27^−^ CD28^+^), as well as the terminally differentiated effector memory cells (TEMRA, CD45RA^+^ CD27^−^ CD28^−^) [39], [40]. Human NK cells can be further divided into the classical cytotoxic NK CD56^dim^ cells (CD56^int^ CD16^+^), and the more immune-regulatory CD56^bright^ (CD56^hi^ CD16) [41]. For staining, PBMCs were thawed, washed twice, and incubated 45 min with the antibody cocktails listed in Table S3. Cells were subsequently washed, re-suspended in 300 µl FACS buffer and analyzed on a FACS Canto flow cytometer (BD Bioscience, San Diego, CA). FACS Diva software version 6.1.3 (BD Bioscience, San Diego, CA) was used to analyze the raw data.

### Cell Culture, Proliferation and ELISA Assays for Cytokine Measurement

PBMCs (10^6^ cells) from each donor were suspended in 500 µl of RPMI (GIBCO, Life technologies, USA) containing 5% FBS (Biochrom AG, Germany), aliquoted into a 48-well plate (Greiner Bio-One Ltd., UK) and cultured under three different conditions: unstimulated, stimulated with anti-CD3 (OKT3, 100 ng/ml) with or without anti-*CD6* mAb (161.8, concentration 10 µg/ml) (Kindly provided by Prof. Francisco Lozano, IDIBAPS, Facultat de Medicina, Universitat de Barcelona, Barcelona). To determine cell proliferation, PBMC from healthy donors were labeled with 2 µM eFluor670 (eBioscience) according to the manufacturer’s protocol. In brief, eFluor stock reconstitution and dilutions were done in PBS at 4°C and incubation with the dye was performed at room temperature (RT) for ten minutes. To stop labeling, standard medium was added and incubated on ice for five minutes. Labeled cells were then transferred into 48-well plates with the respective stimuli for further cell culture. Cells were counterstained with specific lineage markers to assess proliferation at day 0 and 3 by assessing the percentage of cells that had undergone division determined by flow cytometry. The supernatants from the culture were collected on the third day and quantified for the cytokines IL-17 and IFN-γ using Ready-Set-Go ELISA kits (eBiosciences, San Diego, CA, USA). The cytokine concentration (pg/ml) was calculated using the standard curves generated using the respective standards.

### Statistical Analysis of FACS and ELISA Data

ANOVA and two-tailed Student’s-*t* test were used to calculate the differences in surface expression, while differences in proliferation and cytokine production between the different haplotypes were assessed by the non-parametric Mann-Whitney U test using Graphpad Prism software version 5 (GraphPad Software, La Jolla, CA).

## Results

### A *CD6* Haplotype Containing Two Non-synonymous SNPs in the 2^nd^ SRCR Domain is Associated with MS

**Figure 1:**
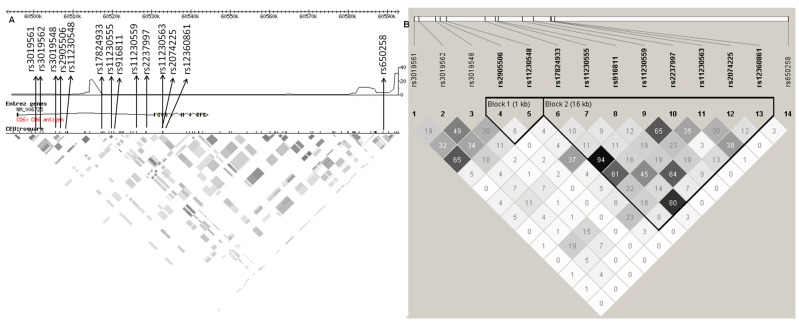
Locations of the 14 Stage 1 SNPs and their linkage disequilibrium (LD) patterns at the *CD6* locus. (A) LD block images with the approximate locations of the 14 SNP markers chosen for this study, recombination spots and LD patterns within the CEU LD plot (Hapmap, version 27) of the *CD6* gene, and (B) LD patterns in the northern Spanish-Basque population using the confidence interval method (Gabriel et al. [54]; generated using the Haploview software).

The first stage of the study was performed on a dataset from northern Spain (Basque Country) constituting a total of 823 MS patients and 814 healthy controls. Nine haplotype tagging (haptag) SNPs (Hapmap release 27) were selected to cover *CD6* gene variability. Three further non-synonymous SNPs were included – rs11230563 (R225W) and rs2074225 (A257V) in the second SRCR domain (Exon 4) and rs12360861 (A271T) in the third SRCR domain (Exon 5). In addition, rs17824933 [5], [18] and rs650258 [17] were also included (Figure 1). Thirteen SNPs are situated in the extracellular domain region, which is divided into two linkage disequilibrium (LD) blocks by a recombination spot with a maximum recombination rate of 21.9 cM/Mb (at position 60514919, Hapmap release #27) (Figure 1), while rs650258 (position 60588858, recombination rate 3.22, Hapmap #27) is located between *CD6* and *CD5* on a recombination peak with maximum rates of 11.12 cM/Mb (at position 60584694,) and 31.4 cM/Mb (at position 60592693) respectively.

Six SNPs were found to be associated with MS susceptibility at nominal significance levels (Table 2), and were replicated in a total of 2265 MS patients and 2600 healthy controls from three different collections (Table 1). One additional non-synonymous SNP rs11230563 (R225W) was also included as it demonstrated a trend towards association (*P* = 0.08, OR = 1.13) in the first-stage screen. Three SNPs emerged with nominal significance from the replication set; rs17824933, rs11230559 and rs2074225 (Table 3). Combined analysis of all datasets showed association of rs11230559, rs2074225, rs17824933 and rs650258.

**Table 2:** Association analysis of 14 *CD6* SNPs in the northern Spanish-Basque dataset.

SNP ID	POWER(1) (%)	BP	LOCATION	AA CHANGE	MINOR/MAJOR ALLELE (2)		HWE *P-*VALUE		FREQ. (3) (4)		*P*-VALUE		OR (95% CI) (3)
							CASES/CNT	CASES/CNT					
rs3019561	96	60500429	Intron 1	–		T/G	0.93/0.92	0.25/0.24		0.44		1.07 (0.91 – 1.25)	
rs3019562	99	60501644	Intron 1	–		C/G	0.67/0.32	0.51/0.47		0.04		1.16 (1.01 – 1.33)	
rs3019548	97	60505515	Intron 1	–		G/A	0.83/0.6	0.40/0.38		0.16		1.11 (0.96 – 1.28)	
rs2905506	95	60506624	Intron 1	–		T/C	0.72/1	0.26/0.25		0.36		1.08 (0.92 – 1.26)	
rs11230548	95	60508145	Intron 1	–		A/C	0.9/0.55	0.86/0.82		0.008		1.28 (1.06 – 1.56)	
rs17824933	94	60517188	Intron 1	–		G/C	0.73/0.09	0.29/0.25		0.007		1.24 (1.06 – 1.45)	
rs11230555	92	60519710	Intron 1	–		A/C	0.84/0.62	0.78/0.77		0.57		1.05 (0.89 – 1.25)	
rs916811	97	60520408	Intron 1	–		A/G	0.46/0.66	0.79/0.72		3.3×10^−5^		1.40 (1.21 – 1.63)	
rs11230559	95	60526110	Intron 1	–		C/T	0.87/0.14	0.30/0.25		0.004		1.25 (1.07 – 1.46)	
rs2237997	98	60528666	Intron 1	–		C/T	0.30/0.015	0.38/0.35		0.07		1.14 (0.99 – 1.32)	
rs11230563	98	60532785	Exon 4	R225W		T/C	0.12/0.82	0.40/0.36		0.08		1.13 (0.98 – 1.31)	
rs2074225	99	60532882	Exon 4	A257V		C/T	0.55/0.94	0.71/0.63		3.1×10^−6^		1.40 (1.21 – 1.63)	
rs12360861	75	60533649	Exon 5	A271T		A/G	0.36/0.43	0.81/0.81		0.79		1.03 (0.85– 1.21)	
rs650258(5)	–	60588858	3′ Intergenic			T/C	0.2/0.06	0.56/0.51		0.0052		1.23 (1.06–1.41)	

(1) Power was calculated with an OR = 1.34 based on the original OR for rs17824933 in the Spanish-Basque dataset [18] using the CEU frequencies (NCBI) for each SNP.

(2) The risk alleles are underlined.

(3) Values represented in terms of the risk allele.

(4) Genotyping success rates were above 95% for all the SNPs typed.

(5) rs650258 was included based on the association observed in a recent genome–wide screen in MS [17].

**Table 3:** Replication and combined analysis of the most significant SNPs.

		REPLICATION			COMBINED	
SNPs (1)	*P*_CMH_	*P*_BD_	OR(2) (95% CI)	*P*_CMH_	*P*_BD_	OR(2) (95% CI)
rs11230548 (A/C)	0.64	0.25	0.97 (0.85–1.1)	0.85	0.17	1.01 (0.90–1.13)
rs17824933 (G/C)	0.02	0.44	1.14 (1.02–1.27)	0.005	0.58	1.16 (1.04–1.28)
rs916811 (A/G)	1.00	0.49	1 (0.89–1.13)	0.45	0.32	1.04 (0.94–1.16)
rs11230559 (C/T)	0.02	0.20	1.14 (1.02–1.27)	0.005	0.31	1.15 (1.05–1.27)
rs11230563 (T/C) (3)	0.77	0.69	0.98 (0.89–1.09)	0.88	0.83	0.99 (0.91–1.09)
rs2074225 (C/T)	0.03	0.53	1.14 (1.01–1.25)	0.0065	0.67	1.14 (1.04–1.25)
rs650258 (T/C)(4)	0.08	0.46	1.085 (0.99–1.19)	0.003	0.30	1.12 (1.04–1.21)

*Abbreviations*: CMH = Cochran-Mantel-Haenszel test, BD = Breslow-Day test, OR = Odds ratio, CI = Confidence interval.

(1) The risk alleles (with respect to the Basque dataset) are underlined.

(2) The ORs are represented for the risk alleles found in the first-stage screen.

(3) rs11230563 was included because it is a non-synonymous SNP that substitutes R225W, and showed a trend towards association in the first stage screen.

(4) rs650258 was included based on the association observed in the Basque dataset.

**Figure 2:**
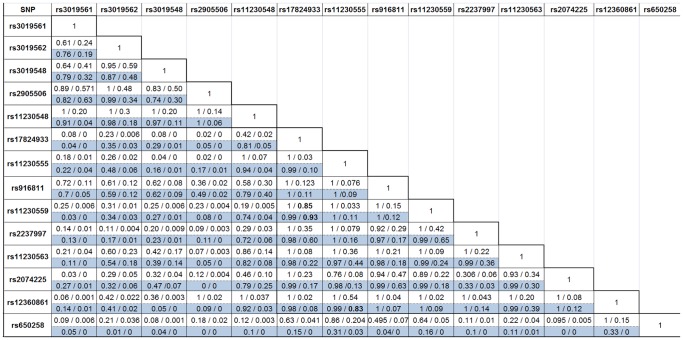
LD values (*D*’/*r^2^* values) between each of the 14 SNP markers genotyped in the Spanish-Basque population. The figure represents a comparison of the LD values between the 14 SNPs genotyped in the northern Spanish-Basque population (*D*’/*r^2^* values) using the values from the CEU 1000 genomes project and from the data generated from the Spanish-Basque population (823 cases/814 controls). The values in white are the *D’*/*r^2^* values from the CEU 1000 Genomes Project, while the blue-shaded ones are generated for the northern Spanish-Basque dataset using Haploview software (version 4.2).

Analysis of LD patterns revealed that rs11230559 is in strong LD with rs17824933 SNP in the northern Spanish dataset (*D’* = 0.99, *r^2^ = *0.93; Figure 2), and also in the combined dataset (not shown), in agreement with data from the 1000 Genomes Project (*D’* = 1, *r^2^* = 0.85; Figure 2). Both these SNPs are in strong LD with two non-synonymous SNPs – rs11230562 (T217M) in the SRCR domain 2 (Exon 4) (*D’* = 1, *r^2^* = 0.94 with rs17824933 and *D’* = 1, *r^2^* = 0.8 with rs11230559) and rs2074233 (G606S) in the cytoplasmic domain (Exon 11) (*D’* = 0.94, *r^2^* = 0.84 with rs17824933 and *D’* = 1, *r^2^* = 0.898 with rs11230559). However, rs650258 showed limited or no LD with any of the three non-synonymous SNPs rs11230563 (*D’* = 0.224, *r^2^* = 0.038), rs2074225 (*D’* = 0.095, *r^2^* = 0.005) and rs12360861 (*D’* = 1, *r^2^* = 0.149) (http://www.broadinstitute.org/mpg/snap/ldsearch.php) [42].

**Figure 3:**
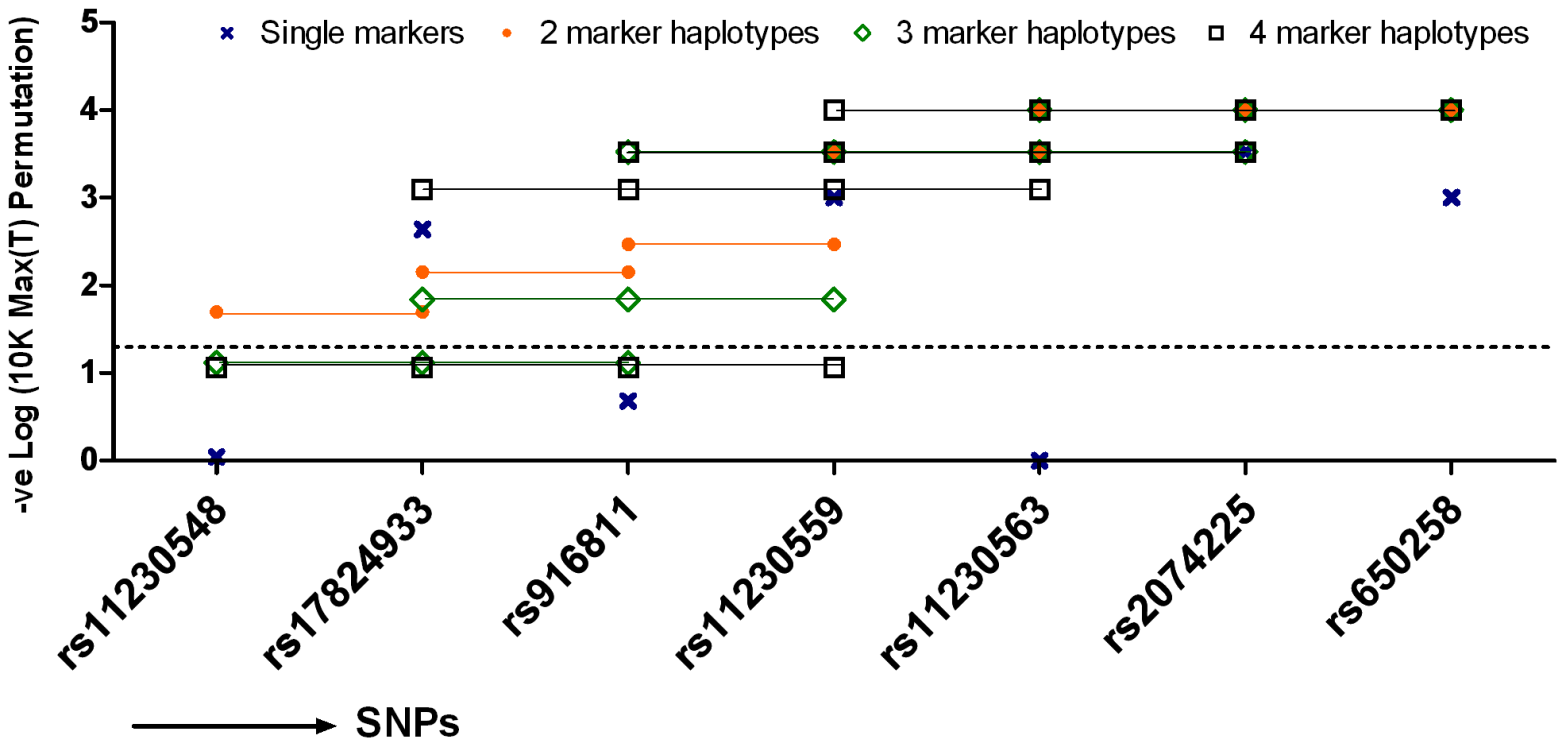
Sliding window haplotype analysis using (A) two and (B) three markers by means of max(T) permutation analysis. A sliding window analysis tests the overall association of the two/three markers that are located adjacent to each other shifting one marker at a time. The analysis was done using the combined dataset containing 3088 cases/3414 controls from the three replication sample sets and the original dataset for the six SNPs selected for replication.

Haplotype analysis was performed on all datasets. A sliding window analysis using two, three and four markers was done followed by 10K max(T) permutation analysis. Furthermore, two- and three-marker analysis using at least one of the two non-synonymous SNPs was also performed (Table S4). The max(T) permutation performs multiple correction based on the number of SNPs tested, while taking account of their correlation (LD) structure. The sliding window analysis showed stronger association (OMNIBUS) of combinations including both the exonic non-synonymous SNPs rs11230563 and rs2074225 (P _max(T) permutation_ = 1×10^−4^), and of rs2074225 and rs650258 (P _max(T) permutation_ = 1×10^−4^) (Figure 3, Table S4). Addition of rs17824933 or rs11230559 to the haplotype markers rs11230563-rs2074225 did not significantly alter the haplotype frequencies (Table S5) or increase the strength of association (*data not shown*), indicating that the association was in essence explained by the haplotype rs11230563-rs2074225 (P _max(T) permutation_ = 1×10^−4^) (Figure 3, Table S4). The use of aggressive tagging on two-marker and three-marker haplotypes showed that rs11230563 could be tagged by markers rs916811 and/or rs11230559 along with rs2074225 (*r^2^* = 0.946) and also by rs916811 and/or rs17824933 with rs2074225 (*r^2^* = 0.892). Similarly, the SNP rs2074225 was tagged by the markers rs11230563 and rs11230559/rs17824933 (*r^2^* = 0.937/0.882 respectively). This indicated that the two non-synonymous SNPs could be tagged by the intronic SNPs in combination with either of the non-synonymous SNP.

A logistic regression analysis was performed assuming additive affects of allele dosage to test for independent effects. The analysis revealed independent effects exerted by the SNPs rs2074225 and rs650258, and conditioning rs11230563 with rs2074225 showed strong additive effects with *P* = 6.2×10^−7^ (Table S6).

In summary, we identified two novel SNPs associated with MS risk, that is, rs11230559 located in the intronic region, and rs2074225, a non-synonymous SNP located in the 2^nd^ SRCR domain, and validated the association of rs650258 [17] with susceptibility to MS. Sliding window haplotype analysis revealed strong association of the two domain-2 (Exon-4) non-synonymous SNPs, which was confirmed by logistic regression analysis.

### The rs11230563-rs2074225 *CD6* Haplotype Modifies CD6 Expression on CD4^+^ and CD8^+^ Naïve T Cells

**Figure 4:**
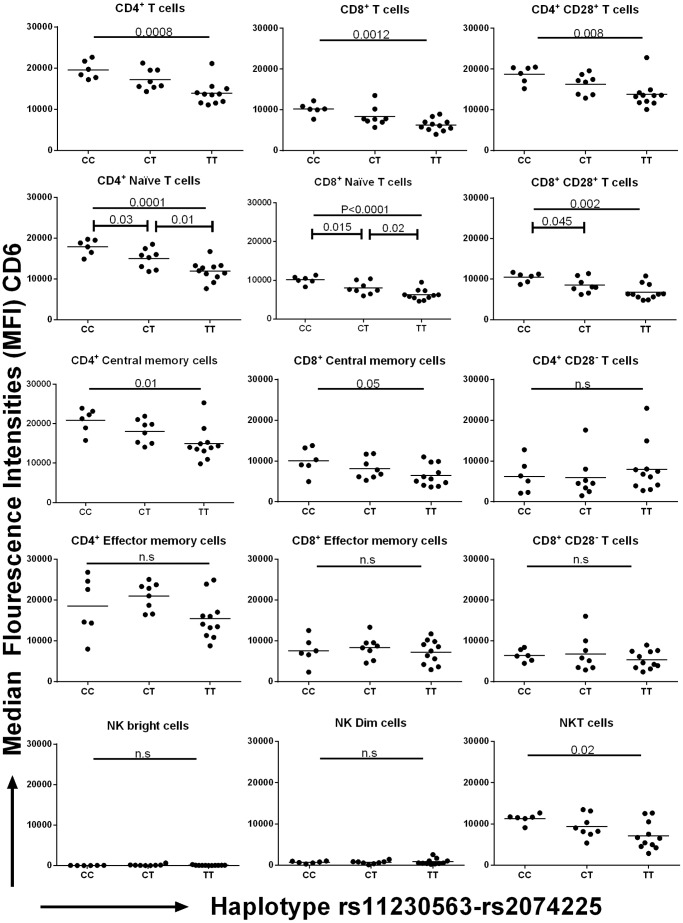
CD6 expression on different cell types segregated by non-synonymous CD6 haplotypes, as analyzed by FACS. A total of 27 PBMC samples representing each of the three haplotypes from the two non-synonymous SNPs rs11230563 and rs2074225 were analysed for CD6 expression on the different cell subsets. The y-axis represents the median fluorescence intensity (MFI) while the x-axis represents the three different haplotypes; i.e. the CC haplotype that confers protection, CT associated with risk and TT conferring mild risk. The distinct subpopulations were identified using different surface markers as listed in Table S4. The markers CD3 and CD56 were used to identify T cells (CD4+/CD8+ CD3^+^ CD56^−^), NK (CD3^−^ CD56^+^), and NKT (CD3^+^ CD56^+^) cell subsets; CD45RA, CD27 and CD28 were used to distinguish naïve (CD45RA^+^ CD27^+^ CD28^+^), central memory cells (CD45RA^−^ CD27^+^) and effector memory cells (CD45RA^−^ CD27^−^ CD28^+^), as well as the terminally differentiated effector memory cells (TEMRA, CD45RA^+^ CD27^−^ CD28^−^); while CD56 and CD16 were used to identify the NK CD56dim (CD56^int^ CD16^+^) and the CD56bright (CD56^hi^ CD16) cells.

FACS analysis was performed on lymphocytes of MS patients genotyped for both *CD6* markers SNPs rs11230563 and rs2074225. CD6 was expressed at very high levels on all CD4^+^ T cell subsets, and to a lesser extent on CD8^+^ and NKT cells (Figure 4, Figure S2). In these three lymphocyte subsets, CD6 expression levels varied significantly, – with the protective haplotype (rs11230563-rs2074225) CC expressing higher CD6 levels than both the risk haplotype CT and the TT haplotype that provides mild risk (Figure 4). These differences were prominent among the CD4^+^ (P_ANOVA_ = 0.0008) and CD8^+^ (P_ANOVA_ = 0.001) lymphocytes, but were also observed in NKT cells (P_ANOVA_ = 0.02). Upon separation of the CD4^+^ and CD8^+^ cell subsets according to their naïve, memory or effector phenotypes, we found the greater differences in the naïve (CD4^+^ naïve cells, *P*_ANOVA_ = 0.0001; CD8^+^ naïve cells, *P*_ANOVA_ <0.0001), and the CD4^+^ central memory subset (*P*_ANOVA_ = 0.01), while no differences were found in the effector subsets and in the terminally differentiated effector cells (CD27^−^ CD28^−^) cells (Figure 4). Of note, NKT cells also expressed CD6, and the haplotype-related differences were also observed in this population (*P*_ANOVA_ = 0.02). NK cells expressed much lower levels of CD6 on their surface, and no significant changes in expression were observed in relation to the CD6 haplotypes. Similar observations were made when the non-synonymous SNPs were analyzed individually (Figure S3, S4).

### Haplotype-specific Differences in Proliferation and Cytokine Secretion upon T-cell Stimulation

The differential expression of CD6 from the different genotypes and haplotypes points to a possible effect on T cell function. Further experiments were aimed at identifying differences in proliferation and in cytokine production. Polyclonally stimulated T cells were assessed to determining whether co-stimulation of CD6 using specific antibodies had a haplotype-related effect on proliferation and on cytokine production. For this purpose, PBMCs were stimulated with anti-CD3 in the presence or absence of anti-CD6 161.8 mAb. Our data showed no significant differences in proliferation and in the levels of IFN-γ and IL-17A secretion between the haplotypes or different conditions (Figure S5). However, even though not significant, donors with the CC haplotype showed relatively higher proliferation and higher levels of IL-17A than those with the CT haplotype. Furthermore, co-stimulation with the anti–CD6 mAb, 161.8 showed a mild decrease in proliferation and IL-17A production (Figure S5).

## Discussion

In this study, we aimed to identify the most important MS-associated *CD6* SNP variants through a fine-mapping exercise. Our data showed a strong association of a non-synonymous SNP rs2074225 with susceptibility in the Spanish-Basque dataset that was replicated in the combined dataset (Table 2, 3). Furthermore, the analysis also revealed association of rs17824933 and rs11230559, both of which are in strong LD with each other (*r^2^*>0.8) and with two other non-synonymous SNPs located in exon 4 (rs11230562) and exon 11 (rs2074233). The association of these SNPs was observed to be stronger in the original dataset (Spanish-Basque residents) when compared to the replication sets; and this may be related to factors such as disease heterogeneity, and the relatively high degree of genetic homogeneity in the Spanish Basque geographic sampling area [43].

Haplotype analysis using sliding window and two-marker approaches implicated a role of two non-synonymous SNPs rs11230563 (R225W) and rs2074225 (A257V). We analyzed expression changes of CD6 on different immune cell types according to genotypes for the individual non-synonymous SNPs genotypes or their haplotypes. Our screen included, apart from the CD4^+^ and CD8^+^ T-cells studied by Kofler et al., [33], also NK cells, NKT cells, and T-effector cell subsets in an attempt to identify the cell type more strongly influenced by the *CD6* non-synonymous SNP genotype/haplotype. Since the mAb used in this study for FACS targets the SRCR domain 1 region, discrimination between different isoforms was not possible. Our data showed a more pronounced effect of the rs11230563-rs2074225 haplotype compared to the individual non-synonymous SNPs (Figure 4, Figure S3, S4). The expression trends showed the protective haplotype (CC) to yield higher surface expression of CD6 when compared to the risk haplotypes (CT and TT), which is in agreement with both the trends observed by Kofler et al. on the full length isoform [33] and the analysis of Heap et al., [44] who reported allele imbalances of eight SNPs in the *CD6* region including rs2074225, rs11230562, and - as inferred though LD patterns, rs17824933.

We also observe that the expression of CD6 from the TT (mild risk) haplotype cells was lower than that from the CT (risk) haplotype. While the CT haplotype is intermediate in terms of CD6 expression between the protective (CC) and mild risk (TT) haplotype in the CD4^+^ and CD8+ T cells, its expression shows a non-significant trend to be higher in CD4+ and CD8+ effector memory T cells. Thus, our data suggests that this CD6-conferred genetic risk is complex and may result in changes in both CD6 expression levels and ligand binding or signal transduction via the non-synonymous SRCR domain 2 SNPs rs11230563 (R225W) and rs2074225 (A257V) in addition to the cytoplasmic domain SNP rs2074233 (G606S).

The contribution of the isoform lacking the ligand-binding domain (CD6ΔD3), of which expression is inversely correlated with that of the full-length form [33], should be factored in into the elucidation of CD6 function. Kofler et al., [33] observed no significant differences in CD6 expression of the full length form between genotypes, but reported increased relative expression of CD6ΔD3 in both CD4^+^ and CD8^+^ T-cells in individuals homozygous for the risk allele rs17824933^GG^. In our study, the naïve CD4^+^ and CD8^+^ T-cell populations were more strongly affected by the *CD6* haplotype than effector and central memory cells. This is important as CD6 is highly expressed in thymic cells and is thought to aid positive selection and provide resistance to apoptosis [20]. Furthermore, Singer et al. [20] and Castro et al. [25] showed the two isoforms to be more highly expressed on mature cells. In the thymus, CD6ΔD3 is under-expressed in the double-positive cells, while the expression of the full-length form is favored which contributes to their survival.

In addition, being a member of the scavenger family, both the isoforms of CD6 appear to bind to bacteria, and may act therefore in an early infectious phase putatively associated with onset of MS, even if the precise mechanism is as yet unknown. Binding studies with LPS and anti-CD6 mAbs showed phosphorylation of ERK1/2 and p38 MAPK indicating activation of the MAPK pathway [45], [46]. Observation of proliferation and cytokine secretion patterns showed a trend towards reduced proliferation by the risk haplotype when compared to the protective haplotype. These findings are in league with the findings of Kofler et al. [33], who observed reduced cell proliferation among the donors with the risk genotype rs17824933^GG^. Similar trends to that of proliferation were observed with IL-17, where higher levels of this cytokine were observed with the protective haplotype (CC) than with the risk haplotype (CT) (not-significant trend; Figure S5). Since addition of anti-CD6 did not significantly alter the proliferation rate and the production of cytokine, it could be inferred that the anti-CD6 mAb could lead to generalized blocking of proliferation and cytokine production and is not SNP-dependent. The IL-17 trends, though inconclusive, do suggest that allelic variation in CD6 may be associated with altered cytokine secretion from Th17 cells. Given the capability of CD6 to bind to bacteria/LPS [45], and the evidence for a role of IL-17A in mediating protection against various pathogens [47], [48], this may be of relevance to the concept of infectious agents acting as triggers for MS. Alternatively, observation of higher IL-17A levels among those with the protective haplotype could indicate a protective/anti-inflammatory role of IL-17A. The ability of IL-17A to protect against development of autoimmune uveitis and ulcerative colitis [49], [50] has been demonstrated; however, this contrasts with the reported disease-promoting role of Th17 cells in MS [51]–[53], and will need further clarification.

Taken together, our results indicate that non-synonymous polymorphic variations in the 2^nd^ SRCR domain are associated with functional changes. Screening of the different cell types showed the most significant expression differences in CD4^+^ and CD8^+^ naïve cells, suggesting that phenotypic expression of CD6 variation may affect the early stages of cell-mediated immune responses.

## Supporting Information

Figure S1 Plots showing Sequenom-based clustering of the alleles from the dataset of Bilbao. Each of the axes represents an allele and each sample in the graph is represented as a dot. Samples homozygous for any of the alleles fall near the x or y-axis while the heterozygotes lie in the graph area between the two axes.
(TIF)

Figure S2 FACS-gating strategy. PBMCs were stained with anti-CD3, CD56, CD16, CD4, CD8 and CD6 antibodies. After gating on lymphocytes, the T and NK cell subsets were defined as shown in the top left panel. From the NK cells, NKbright and NKdim cells were identified according to the level of CD56 and the presence of CD16. T-helper and T cytotoxic lymphocytes were identified by CD4 and CD8 staining, respectively. In these two populations, CD45RA and CD27 were used to define naive, effector and memory cell subsets, as indicated in the two top right panels. The lower panels show the corresponding histograms depicting the expression of CD6 in each of the forementioned subsets. NK histogram, solid line indicates NKbright and dashed line NKdim cells; T cells, solid line indicates CD4 and dashed line CD8 cells; in the CD4 and CD8 histograms, solid line indicates naive cells (N), dotted line effector memory cells (EM) and dashed line central memory cells (CM).
(TIF)

Figure S3 Comparison of CD6 expression on the different cell types with respect to rs11230563 genotypes.
(TIF)

Figure S4 Comparison of CD6 expression on the different cell types with respect to rs2074225 genotypes.
(TIF)

Figure S5 Comparison of proliferation and cytokine levels (pg/ml) between the three haplotypes. PBMCs representing each of the haplotypes (CC = protective, CT = high risk, TT = mild risk) from the healthy donors were cultured in a 48-well plate for three different stimulatory conditions – unstimulated, OKT3 stimulated (100 ng/ml) with/out anti-CD6 161.8 (10 µg/ml). Proliferation was assessed by measuring 10^6^ cells stained with eFluor670 on the third day of culture. The supernatant collected from the three-day culture was used to quantify IL-17A and IFN-γ using ELISA. Each column represents the mean values of the samples. Comparison of proliferation rates and cytokine production was done by the non-parametric Mann-Whitney-U test using Graphpad software (version 5).
(TIF)

Table S1 Demographic and clinical variables of the PBMC samples collected from MS patients for the functional study.
(DOC)

Table S2 Details of the monoclonal antibodies used in flow cytometry.
(DOC)

Table S3 The combination of monoclonal antibodies used for cell surface staining.
(DOC)

Table S4 OMNIBUS association *P*-values of haplotypes generated using sliding window or multiple marker analysis on the merged datasets.
(DOC)

Table S5 Comparison of the haplotype frequencies in the four datasets of (A) the 2 associated NS haplotype SNP markers, or (B) three NS SNP markers.
(DOC)

Table S6 Logistic regression analysis on the combined dataset showing additive effects of the SNPs when conditioned to rs11230563, rs2074225 and rs650258.
(DOC)
