# Supplementary figures and images for: Fine Mapping and Functional Analysis of the Multiple Sclerosis Risk Gene CD6
Source: PLoS One. 2013 Apr 24;8(4):e62376. doi: 10.1371/journal.pone.0062376 (PMC3634811; doi:10.1371/journal.pone.0062376)

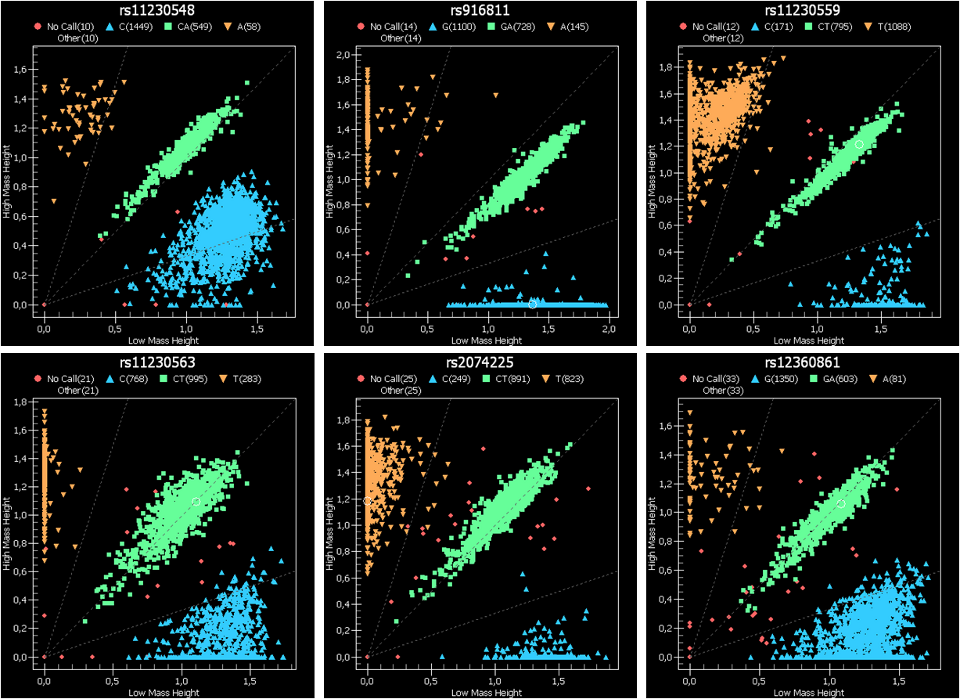

Supplement: Figure S1 — Plots showing Sequenom-based clustering of the alleles from the dataset of Bilbao. Each of the axes represents an allele and each sample in the graph is represented as a dot. Samples homozygous for any of the alleles fall near the x or y-axis while the heterozygotes lie in the graph area between the two axes. (TIF) [file pone.0062376.s001.tif]

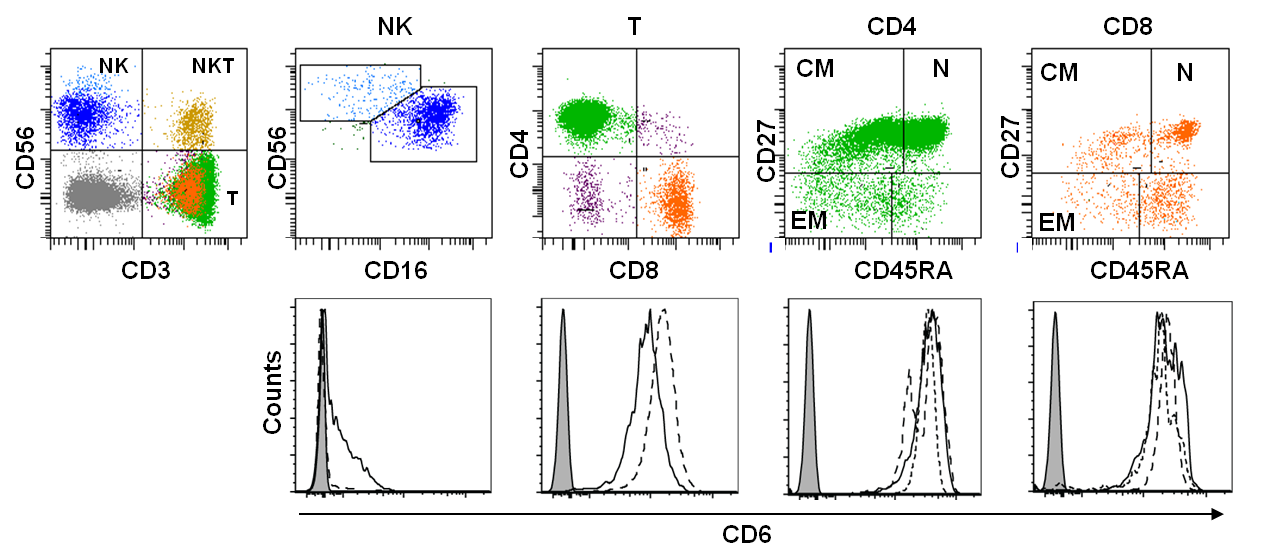

Supplement: Figure S2 — FACS-gating strategy. PBMCs were stained with anti-CD3, CD56, CD16, CD4, CD8 and CD6 antibodies. After gating on lymphocytes, the T and NK cell subsets were defined as shown in the top left panel. From the NK cells, NKbright and NKdim cells were identified according to the level of CD56 and the presence of CD16. T-helper and T cytotoxic lymphocytes were identified by CD4 and CD8 staining, respectively. In these two populations, CD45RA and CD27 were used to define naive, effector and memory cell subsets, as indicated in the two top right panels. The lower panels show the corresponding histograms depicting the expression of CD6 in each of the forementioned subsets. NK histogram, solid line indicates NKbright and dashed line NKdim cells; T cells, solid line indicates CD4 and dashed line CD8 cells; in the CD4 and CD8 histograms, solid line indicates naive cells (N), dotted line effector memory cells (EM) and dashed line central memory cells (CM). (TIF) [file pone.0062376.s002.tif]

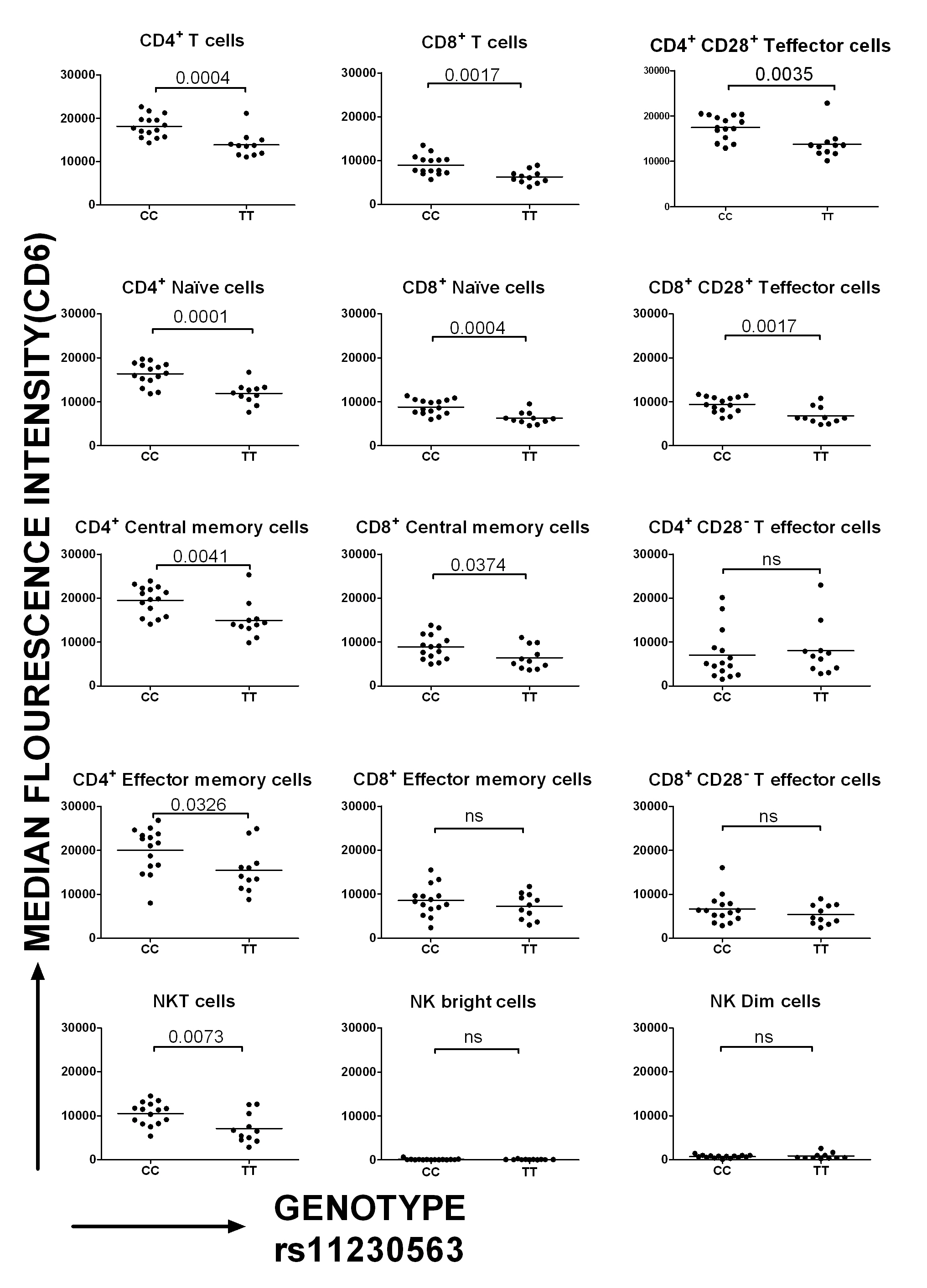

Supplement: Figure S3 — Comparison of CD6 expression on the different cell types with respect to rs11230563 genotypes. (TIF) [file pone.0062376.s003.tif]

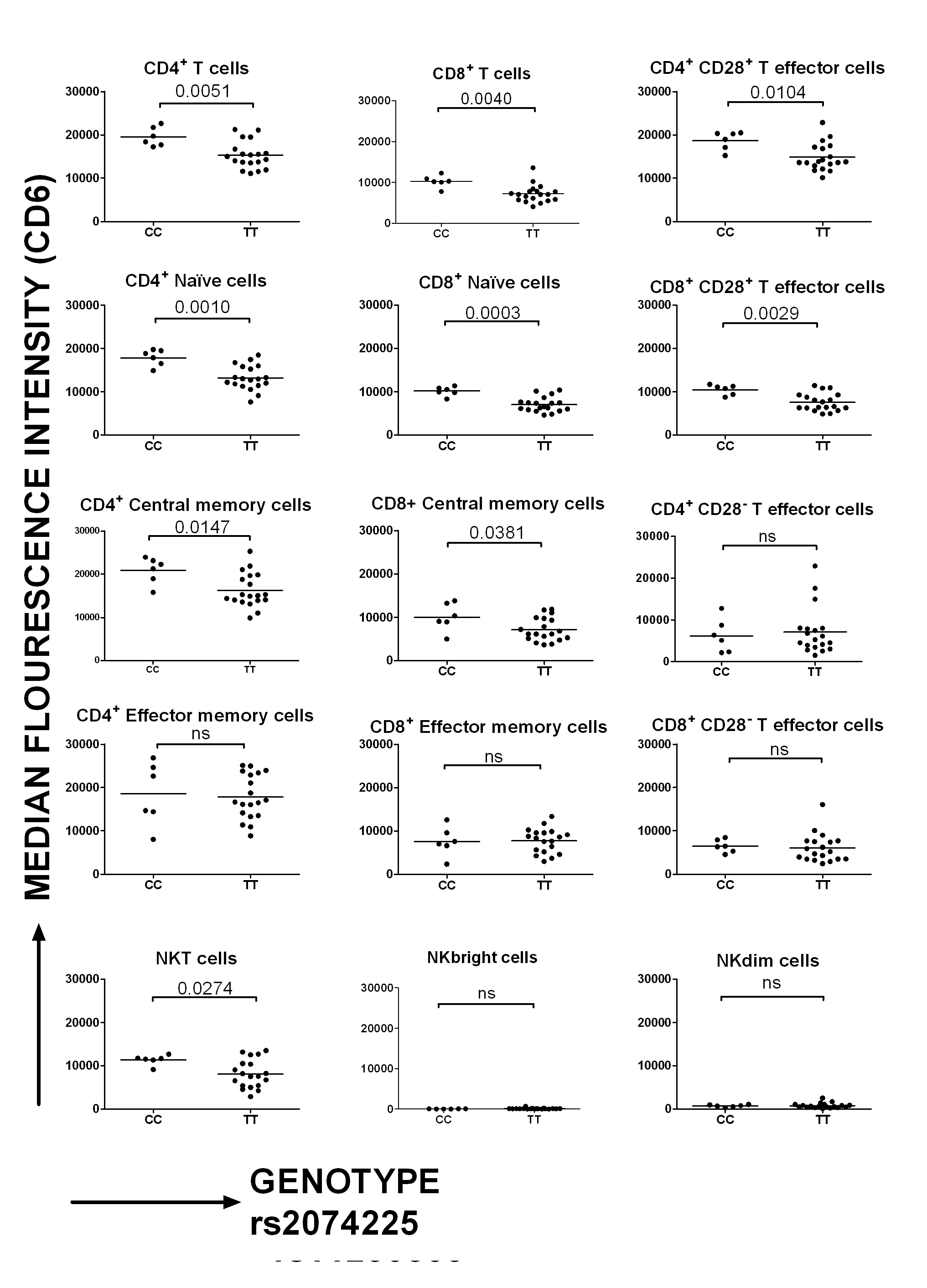

Supplement: Figure S4 — Comparison of CD6 expression on the different cell types with respect to rs2074225 genotypes. (TIF) [file pone.0062376.s004.tif]
