# Supplementary material for: Fine Mapping and Functional Analysis of the Multiple Sclerosis Risk Gene CD6
Source: PLoS One. 2013 Apr 24;8(4):e62376. doi: 10.1371/journal.pone.0062376 (PMC3634811; doi:10.1371/journal.pone.0062376)
Supplement: Table S1 — Demographic and clinical variables of the PBMC samples collected from MS patients for the functional study. (DOC) [file pone.0062376.s006.doc]

**Table S1**. Demographic and clinical variables of the PBMC samples collected from MS patients for the functional study.

| **Characteristics** | **Statistics** | |
| --- | --- | --- |
| Gender | MS Patients | Healthy Donors |
| Female / Male ratio (%) | 18/9 (66.6/33.3) | 7/5 (58.3/41.6) |
| Age in years | 43.1 ± 11.9 (23-66) | 33.08 ± 6.68 (25-45) |
| Present clinical form (%) | | |
| RR-MS | 23 (85.2) | |
| SP-MS | 4 (14.8) | |
| Mean age at onset in years | 27.9 ± 10.4 (15-55) | |
| Mean disease duration in years | 15.1 ± 6.6 (5-28) | |
| Current EDSS Score | 2.22 ± 1.21 (0-5) | |

*Abbreviations*: RR-MS = Relapsing-remitting form of multiple sclerosis, PP-MS = primary progressive
