# Supplementary material for: Fine Mapping and Functional Analysis of the Multiple Sclerosis Risk Gene CD6
Source: PLoS One. 2013 Apr 24;8(4):e62376. doi: 10.1371/journal.pone.0062376 (PMC3634811; doi:10.1371/journal.pone.0062376)
Supplement: Table S2 — Details of the monoclonal antibodies used in flow cytometry. (DOC) [file pone.0062376.s007.doc]

**Table S2. Details of the monoclonal antibodies used in flow cytometry.**

| **mab Specificity** | **Fluorescent label** | **Clone** | **Type** | **Company** |
| --- | --- | --- | --- | --- |
| **CD3** | PeCy5.5 | UCHT1 | primary | Biozol |
| **CD8a** | PacBlue | RPA-T8 | primary | Biolegend |
| **CD4** | PECy7 | RPA-T4 | primary | BD Biosciences |
| **CD4** | AF488 | RPA-T4 | primary | BD Biosciences |
| **CD56** | APC | N901 (NKH-1) | primary | Beckman Coulter |
| **CD16** | APCCy7 | 3G8 | primary | Biolegend |
| **CD28** | PECy7 | CD28.2 | primary | Biolegend |
| **CD27** | APCCy7 | M-T271 | primary | BD Biosciences |
| **CD45RA** | APC | HI100 | primary | eBiosciences |
| **CD6 (D1)** | PE | M-T605 | primary | BD Biosciences |
| **161.8** | Unlabelled | Received from Prof. Francisco Lozano, IDIBAPS, Facultat de Medicina, Universitat de Barcelona, Barcelona. | | |
