# Supplementary material for: Fine Mapping and Functional Analysis of the Multiple Sclerosis Risk Gene CD6
Source: PLoS One. 2013 Apr 24;8(4):e62376. doi: 10.1371/journal.pone.0062376 (PMC3634811; doi:10.1371/journal.pone.0062376)
Supplement: Table S3 — The combination of monoclonal antibodies used for cell surface staining. (DOC) [file pone.0062376.s008.doc]

**Table S3.** The combination of monoclonal antibodies used for cell surface staining.

| Cell Subset | Fluorescent Dye | | | | | | |
| --- | --- | --- | --- | --- | --- | --- | --- |
|  | V450 | Alexa Flour 488 | PE | PE-Cy5.5 | PE-Cy7 | APC | APC-Cy7 |
| Unstained | – | – | – | – | – | – | – |
| FMO Control | CD8 | – | – | CD3 | CD4 | CD56 | CD16 |
| T-Effector | CD8 | – | CD6 | CD3 | CD28 | CD45RA | CD27 |
| Natural Killer | CD8 | – | CD6 | CD3 | CD4 | CD56 | CD16 |
