# Supplementary material for: Fine Mapping and Functional Analysis of the Multiple Sclerosis Risk Gene CD6
Source: PLoS One. 2013 Apr 24;8(4):e62376. doi: 10.1371/journal.pone.0062376 (PMC3634811; doi:10.1371/journal.pone.0062376)
Supplement: Table S4 — OMNIBUS association P-values of haplotypes generated using sliding window or multiple marker analysis on the merged datasets. (DOC) [file pone.0062376.s009.doc]

**Table S4.** OMNIBUS associations *P*-values of haplotypes generated using sliding window or multiple marker analysis on the merged datasets.

| NSNP | NHAP | CHR | SNPs | STAT | P | EMP1 | EMP2 |
| --- | --- | --- | --- | --- | --- | --- | --- |
| 1 | 2 | 11 | rs11230548 | 0.973 | 0.324 | 0.3228 | 0.90 |
| 1 | 2 | 11 | rs17824933 | 13 | 0.0003 | 0.0002 | 0.002 |
| 1 | 2 | 11 | rs916811 | 4.63 | 0.0315 | 0.0293 | 0.21 |
| 1 | 2 | 11 | rs11230559 | 14.4 | 0.000144 | 1 × 10^-4^ | 0.001 |
| 1 | 2 | 11 | rs11230563 | 0.278 | 0.598 | 0.605 | 0.995 |
| 1 | 2 | 11 | rs2074225 | 18.5 | 1.73 × 10^-5^ | 1 × 10^-4^ | 0.0003 |
| 1 | 2 | 11 | rs650258 | 14.5 | 0.00014 | 1 × 10^-4^ | 0.001 |
| 2 | 4 | 11 | rs11230548 - rs17824933 | 14.2 | 0.0026 | 0.002 | 0.020 |
| 2 | 3 | 11 | rs17824933 - rs916811 | 14 | 0.001 | 0.0005 | 0.007 |
| 2 | 3 | 11 | rs916811 - rs11230559 | 15.5 | 0.0004 | 0.0004 | 0.0034 |
| 2 | 3 | 11 | rs11230559 - rs11230563 | 22.7 | 1.21 × 10^-5^ | 1 × 10^-4^ | 0.0003 |
| 2 | 3 | 11 | rs11230563 - rs2074225 | 24.1 | 5.76 × 10^-6^ | 1 × 10^-4^ | 1 × 10^-4^ |
| 2 | 4 | 11 | rs2074225 - rs650258 | 33.4 | 2.67 × 10^-7^ | 1 × 10^-4^ | 1 × 10^-4^ |
| 3 | 6 | 11 | rs11230548 - rs17824933 - rs916811 | 15 | 0.0103 | 0.0085 | 0.0768 |
| 3 | 4 | 11 | rs17824933 - rs916811 - rs11230559 | 15 | 0.002 | 0.0017 | 0.0145 |
| 3 | 4 | 11 | rs916811 - rs11230559 - rs11230563 | 24.1 | 2.37 × 10^-5^ | 1 × 10^-4^ | 0.0003 |
| 3 | 4 | 11 | rs11230559 - rs11230563 - rs2074225 | 25.1 | 1.51 × 10^-5^ | 1 × 10^-4^ | 0.0003 |
| 3 | 6 | 11 | rs11230563 - rs2074225 - rs650258 | 38.7 | 2.7 × 10^-7^ | 1 × 10^-4^ | 1 × 10^-4^ |
| 4 | 6 | 11 | rs11230548 - rs17824933 - rs916811 - rs11230559 | 14.7 | 0.012 | 0.011 | 0.086 |
| 4 | 5 | 11 | rs17824933 - rs916811 - rs11230559 - rs11230563 | 24 | 7.9 × 10^-5^ | 0.0004 | 0.0008 |
| 4 | 5 | 11 | rs11230559 - rs11230563 - rs2074225 - rs2074225 | 27.9 | 1.3 × 10^-5^ | 1 × 10^-4^ | 0.0003 |
| 4 | 6 | 11 | rs11230559 - rs11230563 - rs2074225 - rs650258 | 38.2 | 3.4 × 10^-7^ | 1 × 10^-4^ | 1 × 10^-4^ |
