# Supplementary material for: Fine Mapping and Functional Analysis of the Multiple Sclerosis Risk Gene CD6
Source: PLoS One. 2013 Apr 24;8(4):e62376. doi: 10.1371/journal.pone.0062376 (PMC3634811; doi:10.1371/journal.pone.0062376)
Supplement: Table S5 — Comparison of the haplotype frequencies in the four datasets of (A) the 2 associated NS haplotype SNP markers, or (B) three NS SNP markers. (DOC) [file pone.0062376.s010.doc]

**Table S5.** Comparison of the haplotype frequencies in the four datasets of (A) the 2 associated NS haplotype SNP markers, or (B) three NS SNP markers.

(A)

| SNP | rs11230563 (C > T) | rs2074225 (C > T) | Frequency Cases / Controls | | | |
| --- | --- | --- | --- | --- | --- | --- |
|
| POSITION OF AA | R225W | A257V | Spanish-Basque | Madrid | Andalucía | American Whites |
| COMBINATION 1 | R | A | 0.295 / 0.37 | 0.30 / 0.31 | 0.29 / 0.31 | 0.36 / 0.40 |
| COMBINATION 2 | R | V | 0.31 / 0.26 | 0.291 / 0.29 | 0.31 / 0.28 | 0.31 / 0.26 |
| COMBINATION 3 | W | V | 0.40 / 0.37 | 0.41 / 0.40 | 0.395 / 0.40 | 0.34 / 0.34 |

**(B)**

| SNP | rs11230562 (C > T)* | rs11230563 (C > T) | rs2074225 (C > T) | Frequency Cases / Controls | | | |
| --- | --- | --- | --- | --- | --- | --- | --- |
|
| POSITION OF AA | T217M | R225W | A257V | Spanish-Basque | Madrid | Andalucía | American Whites |
| COMBINATION 1 | T | R | A | 0.295 / 0.37 | 0.30 / 0.31 | 0.29 / 0.31 | 0.36 / 0.40 |
| COMBINATION 2 | M | R | V | 0.29 / 0.25 | 0.27 / 0.26 | 0.28 / 0.25 | 0.27 / 0.23 |
| COMBINATION 3 | T | W | V | 0.395 / 0.37 | 0.41 / 0.40 | 0.39 / 0.40 | 0.33 / 0.34 |
| COMBINATION 4 | T | R | V | 0.019 / 0.014 | 0.022 / 0.028 | 0.032 / 0.029 | 0.04 / 0.03 |

* rs11230562 was substituted with rs17824933 to estimate the haplotype frequency based on the observation of strong LD (*r^2^* > 0.8)
