# Supplementary material for: Fine Mapping and Functional Analysis of the Multiple Sclerosis Risk Gene CD6
Source: PLoS One. 2013 Apr 24;8(4):e62376. doi: 10.1371/journal.pone.0062376 (PMC3634811; doi:10.1371/journal.pone.0062376)
Supplement: Table S6 — Logistic regression analysis on the combined dataset showing additive effects of the SNPs when conditioned to rs11230563, rs2074225 and rs650258. (DOC) [file pone.0062376.s011.doc]

**Table S6.** Logistic regression analysis on the combined dataset showing additive effects of the SNPs when conditioned to rs11230563, rs2074225 and rs650258.

|  | Conditioned to SNP | | rs11230563 | | rs2074225 | | rs650258 | |
| --- | --- | --- | --- | --- | --- | --- | --- | --- |
| CHR | Locus | Allele | Adding Locus to SNP (P-Value) | Adding SNP to locus (P-Value) | Adding Locus to SNP (P-Value) | Adding SNP to locus (P-Value) | Adding Locus to SNP (P-Value) | Adding SNP to locus (P-Value) |
| 11 | rs11230548 | A | n.s | n.s | n.s | 4.72 × 10^-6^ | n.s | 9.05 × 10^-5^ |
| 11 | rs17824933 | G | 8.81 × 10^-6^ | 0.009 | n.s | 0.002 | 0.003 | 0.001 |
| 11 | rs916811 | G | 0.04 | n.s | 0.02 | 6.04 × 10^-6^ | 0.014 | 2.47 × 10^-5^ |
| 11 | rs11230559 | C | 1.81 × 10^-6^ | 0.005 | 0.05 | 0.002 | 0.001 | 0.002 |
| 11 | rs11230563 | T | — | — | 0.02 | 6.19 × 10^-7^ | n.s | 4.42 × 10^-5^ |
| 11 | rs2074225 | T | 6.19 × 10^-7^ | 0.02 | — | — | 2.60 × 10^-5^ | 1.91 × 10^-5^ |
| 11 | rs650258 | C | 4.42 x 10^-5^ | n.s | 1.91× 10^-5^ | 2.60 × 10^-5^ | — | — |
